# Characterization of enterovirus 71 infection and associated outbreak of Hand, Foot, and Mouth Disease in Shawo of China in 2012

**DOI:** 10.1038/srep38451

**Published:** 2016-12-12

**Authors:** Michelle Y. Liu, Jin Liu, Weijian Lai, Jun Luo, Yingle Liu, Gia-Phong Vu, Zhu Yang, Phong Trang, Hongjian Li, Jianguo Wu

**Affiliations:** 1 College of Life Sciences, Jinan University, Guangzhou, Guangdong 510632, China; 2 State Key Laboratory of Virology, College of Life Sciences, Wuhan University, Wuhan, Hubei 430072, China; 3 School of Public Health, University of California, Berkeley, CA 94720, USA; 4 Taizhou Institute of Virology, Taizhou, Jiangsu 225300, China; 5 Jiangsu Affynigen Biotechnologies, Inc., Taizhou, Jiangsu 225300, China

## Abstract

Infection of enterovirus 71 (EV71) and associated hand, foot, and mouth disease (HFMD) are recognized as emerging public health issues worldwide. Hundreds of thousands of children are annually infected with EV71 and develop HFMD in China alone. Studies of EV71 infection are critical to the treatment and prevention of the associated HFMD outbreaks. In this report, we studied an outbreak of 105 HFMD cases in Shawo Township of China between September to October 2012. More than 90% of cases were children younger than 9 years old, with over 50% of cases aged 3–6 years old. Laboratory studies detected a high prevalence of EV71 and suggested EV71 as the most common enterovirus causing HFMD in Shawo. Sequencing analysis showed that the EV71 strains from Shawo belong to the C4 subgenotype, and are phylogenetically more related to those from the distant city of Nanchang than those from the nearby city of Wuhan with distinct variations. More girls were found to be associated with EV71 in Shawo whereas more boys were associated with EV71 in Wuhan and Nanchang. Our studies further the understanding of the molecular epidemiological features of HFMD and infection by enteroviruses in China.

Hand, foot, and mouth disease (HFMD) is a commonly benign, febrile disease primarily affecting infants and young children and is characterized by ulcers on the mouth, hands, and/or feet^1^,^2^,^3^. HFMD is often associated with enteroviruses, most commonly enterovirus 71 (EV71) and coxsackievirus A16 (CVA16)^4^,^5^. However, several other serotypes of human enteroviruses such as coxsackievirus A4-A10, B2-B5, and Echovirus 18, have been reported to also cause HFMD^4^. The main clinical manifestations of HFMD are fever and rash on the hands, feet, and mouth. Most HFMD cases are mild and self-limited which usually resolving in 5–6 days. However, some EV71-related HFMD cases are severe and even fatal if the virus causes neurological infection (e.g. aseptic meningitis and brainstem encephalitis)^6^,^7^,^8^,^9^. Studying the epidemiology of human enteroviruses is important to control and prevent HFMD.

In recent decades, HFMD has been recognized as an emerging public health issue across the Western Pacific region and especially in mainland China^1^,^2^,^3^. Hundreds of thousands of children develop HFMD each year in addition to recent outbreaks in Shanghai and Zunyi, with over 2,000,000 and 6,000 cases spanning one year, respectively^1^,^2^,^3^. There are also sporadic HFMD outbreaks in East Asia, Southeast Asia and other regions since it was first reported in California, USA in 1969^10^,^11^,^12^. Due to the high frequency and profound effects of enterovirus infections and their associated-HFMD cases, it is important to study and control enterovirus-related HFMD epidemics^5^,^9^. Although EV71 infection appears to be more virulent and causes more fatal cases, CVA16 infection can also result in severe cases^4^,^5^,^13^. Vaccines for EV71 infection have been recently developed. However, the multi-causational viral nature of HFMD highlights the importance of further developing public health prevention methods.

EV71 and CVA16, two members of the enterovirus family, are positive-sense, single stranded (+ssRNA) non-enveloped RNA viruses with excellent transmission ability due to their stability in the environment^4^,^5^. Enteroviruses also replicate with high mutation rates and frequent viral recombination, which can lead to the generation of new viral variants^14^,^15^,^16^,^17^,^18^. It is generally believed that novel viral variants contribute to HFMD outbreaks^5^,^9^. Several researchers have already reported the epidemiology of HFMD and enterovirus infection in the areas including Taiwan, Japan, Hong Kong, the United States, and Europe^19^,^20^,^21^,^22^,^23^,^24^,^25^,^26^,^27^,^28^. Further studies of circulating enterovirus strains and their distribution are critical to our understanding of enterovirus infection and developing new antiviral compounds and novel therapeutic strategies.

In the current study, we reported enterovirus infection and HFMD in the Shawo Township of China from September to October 2012. Shawo is a rural township located in Hubei Province and is about 100 miles from the city of Wuhan. Wuhan, one of the largest cities in China, is also the capital city of Hubei Province which located near the epicenter of the 2008 HFMD epidemic in the city of Fuyang in Anhui Province (Fig. 1). We have recently reported the infection of enteroviruses and HFMD outbreaks in Wuhan and in northern Hubei Province^29^ as well as in the city of Nanchang in the adjacent Jiangxi Province between 2010 and 2011^30^. However, HFMD outbreaks and their associated enterovirus infections in Shawo have not been reported. Equally unknown is the nature of the HFMD outbreaks and enterovirus infection in Hubei Province after 2011.

In the current study, we reported an outbreak of HFMD in the Township of Shawo in 2012. One hundred and five HFMD clinical samples were collected and analyzed. As the results show, most of the reported HFMD cases (90%) were children younger than 9 years old. Laboratory studies detected a high prevalence of EV71 and CVA16 amongst the cases and suggested EV71 as the most common viral agent causing HFMD in Shawo. We also analyzed the genetic characteristics of the partial VP1 gene of EV71 using phylogenetic analysis. Our results suggest that the EV71 strains isolated in Shawo belong to the C4 subgenotype commonly found in China, and are phylogenetically more related to the virus strains from the city of Nanchang in the distant Jiangxi Province than those from the nearby city of Wuhan in Hubei Province with distinct variations. These results further our understanding of the molecular epidemiological features of HFMD and enterovirus infection in China.

## Results

### Design of the study

We focused on an outbreak of HFMD in the Township of Shawo between September 1 and October 28, 2012. During this period, one hundred and five suspected HFMD patients seen at the Shawo People’s Clinic were recorded. Patients were given a thorough medical exam, clinical samples were collected, and a questionnaire/survey was filled out. We then performed laboratory procedures such as quantitative RT-PCR (qRT-PCR) and virus isolating and culture to analyze the samples for the presence of human enteroviruses, including EV71 and CVA16, which are among the most common causes of HFMD. We also carried out epidemiological analyses based on the results of the medical exams, completed surveys, and laboratory experiments.

### Infections of EV71 and CVA16 in HFMD cases in Shawo

Total RNAs were extracted from collected samples and then tested by qRT-PCR with primers, and screened for universal enteroviruses (EVU), EV71, and CVA16, respectively. To further confirm the qRT-PCR results, some of the samples that were tested positive for EV71 were then cultured in RD and Hep-2 cell lines. EV71 viruses were isolated from the cultures. The presence of EV71 VP1 sequence was verified by PCR amplification and sequencing analysis.

Of the 105 suspected HFMD cases, 66 (66/105, 62.9%) were infected with enteroviruses, 48 (48/105, 45.7%) were infected with EV71, and 16 (16/105, 15.2%) were infected with CV16, respectively (Supplemental Information Table S1). Furthermore, 8 (8/105, 7.6%) were co-infected with both EV71 and CVA16. Sixty four of the 66 EVU-positive samples were also found to contain either EV71 (48/66, 72.7%) or CV16 (16/66, 24.2%) (Supplemental Information Table S1). Thus, as the results shown, the majority (62.9%) of HFMD cases in our studies were infected with enteroviruses, mainly EV71 and CVA16. Furthermore, EV71 infection (72.7%) was more common than CVA16 infection (24.2%) among the HFMD patients in Shawo in 2012.

### General characteristics of HFMD cases and infection of enteroviruses in Shawo

**Figure 2:**
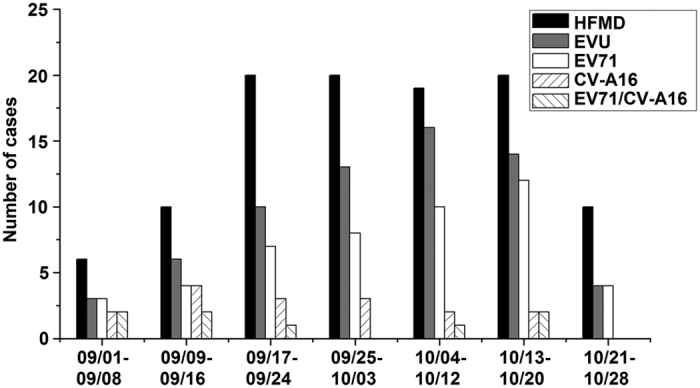
Distribution of the HFMD cases and those containing enteroviruses between September 1 to October 28, 2016. The cases were tested positive for enterovirus (EVU), enterovirus 71 (EV71), and coxsackievirus 16 (CVA16), and positive for both EV71 and CVA16 (EV71/CVA16), respectively.

Between September 1 and October 28 2012, the Shawo People’s Clinic recorded 105 suspected HFMD cases. The HFMD outbreak in Shawo Township spanned a time period of about 8 weeks, the highest peak occurring during the weeks of September 17 to October 20 (Fig. 2). During the following months from November to December, less than 10 HFMD cases were reported at the same clinic.

**Figure 3:**
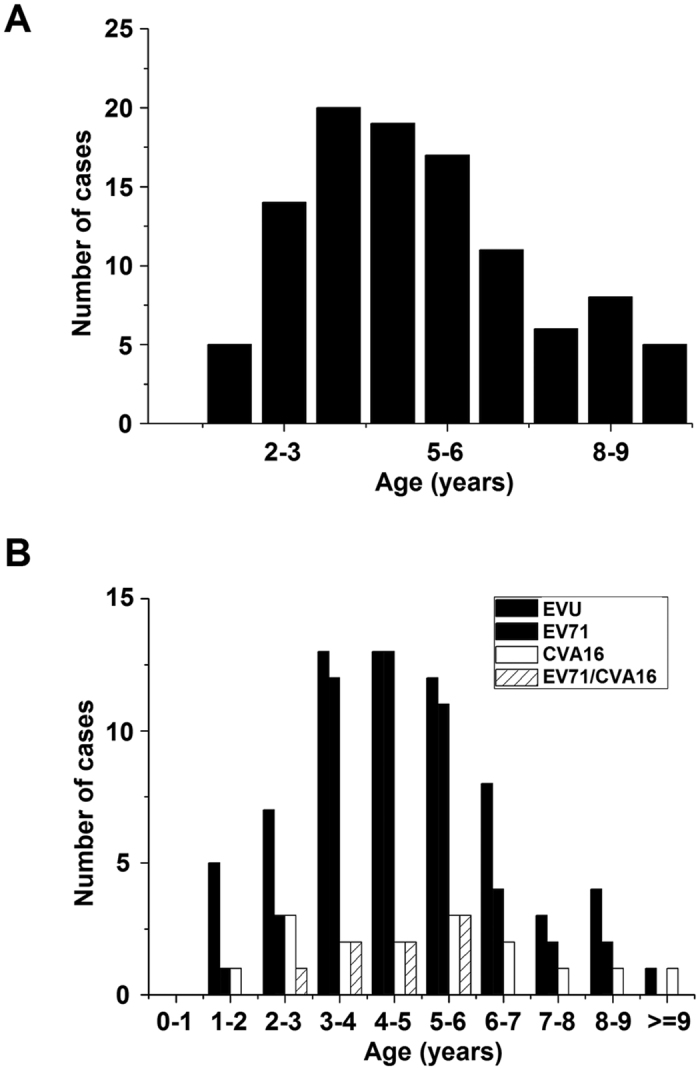
Age distribution of the HFMD cases (**A**) and those containing enteroviruses (**B**). The cases were tested positive for enterovirus (EVU), enterovirus 71 (EV71), and coxsackievirus 16 (CVA16), and for both EV71 and CVA16 (EV71/CVA16), respectively.

Amongst 105 HFMD cases, the majority (92/105, 87.6%) of the cases were children (<8 years old) with 5 cases in patients older than 9 years of age. Those at the peak of numbers of cases were 3 to 5 years old (Fig. 3A). When excluding cases aged older than 8 years old, there was an overall increase incidence of HFMD cases as the age increased from 0 to 4 and an overall decline as the age increased from 4 to 8. Our results indicate that the majority of HFMD cases were children aged below 10 years, as reported previously^5^,^22^,^31^.

The age peak found in the HFMD outbreak of Shawo showed that HFMD occurred most often in children of 3–6 years old (Fig. 3A). Most Chinese children (<3 years old) remain at home under the care of parents, then start attending daycare and kindergarten when they are 3–6 years, and go to elementary school when they are older than 6 years old. Of the HFMD cases, 56 cases (56/105, 53.3%) were 3–6 years old and 49 cases (49/105, 46.7%) were either younger than 3 years or older than 6 years (Fig. 3A). These results suggest that the occurrence of HFMD may be associated with schooling in daycare centers and kindergartens but has little relation with household care or attending elementary school. Thus, schooling in daycare centers/kindergartens may play a role in the transmission of HFMD in Shawo.

EV71-positive cases appeared to increase as HFMD cases increased in the age group of 1 to 4 and decrease as HFMD cases decreased in the age groups of 5 to 8 years old (Fig. 3B), suggesting that EV71 infection followed the pattern of the HFMD outbreak. In contrast, CV16 infection did not correlate as well with HFMD infection patterns. For example, the cases of CV16 infection decreased while the number of HFMD cases increased in the age groups of 2 to 4 (Fig. 3B). This notion is also supported by the presence of EV71 in the samples (45.7% in HFMD and 72.8% in EV71-positive cases, respectively). These observations indicated that more than 60% of the HFMD cases were associated with enterovirus infections, and suggested EV71 as the most common enterovirus causing HFMD in Shawo Township in 2012.

### Gender and symptom distribution of HFMD cases and viral infections

**Figure 4:**
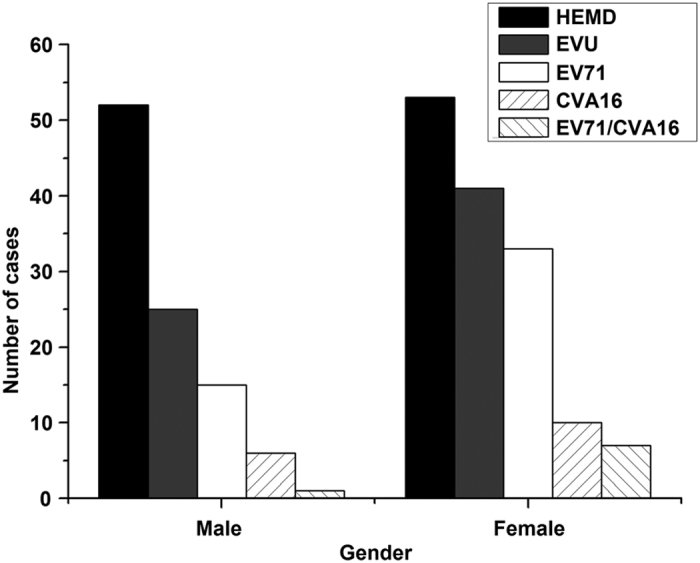
Gender distribution of the HFMD cases and those containing enteroviruses. The cases were tested positive for enterovirus (EVU), enterovirus 71 (EV71), and coxsackievirus 16 (CVA16), and for both EV71 and CVA16 (EV71/CVA16), respectively.

Amongst the HFMD cases, 52 cases (52/105, 49.5%) were boys and 53 cases (53/105, 50.5%) were girls (Fig. 4). Of the EVU cases, 22 cases (22/66, 33.3%) were boys and 44 cases (44/66, 66.7%) were girls. Of the EV71 cases, 15 cases (15/48, 31.3%) were boys and 33 cases (33/48, 68.7%) were girls. Of the CVA16 cases, 6 cases (6/16, 37.5%) were boys and 10 cases (10/16, 62.5%) were girls (Fig. 4). The numbers of male in EVU, EV71, and CVA16 cases were consistently less than the numbers of female in these cases, respectively. Of the 105 cases of HFMD, none of them were fatal. Seventy-nine cases had fever, 38 had cough, and 58 had catarrhal symptoms (Table 1). 82 cases had oral lesions and 64 had vesicles on hand and foot, with the most cases between 3 and 6 years old (Table 1).

### Geographical distribution of HFMD cases in Shawo

Shawo is a rural township composed of 11 villages: Zhaozhai, Baotuan, Huqiao, Pailou, Xinwan, Huangshan, Zouma, Jiajiang, Yuba, Caobei, and Shawo Village (Table 2). Shawo Village is the largest village with the most residents, and also has one of the only two daycare centers/kindergartens in Shawo Township. Of 11 villages in Shawo Township, Shawo Village residents comprised 52 (52/105, 49.5%) cases and all other village residents comprised 1–15 (1.0–14.3%) cases (Table 2).

Most of the HFMD cases that were tested to be positive for the presence of enteroviruses were also found to be associated with EV71 infection. For example, 24 of 30 HFMD cases that were EVU positive in Shawo Village were also positive for EV71 while 3 of 3 HFMD cases that were EVU positive in Huangshan Village were also EV71 positive (Table 2). These results suggested that EV71 represented the major cause of the enterovirus infections associated with HFMD cases in Shawo Township. For further investigation of these cases, field trips were made to Shawo Township. Analysis of the HFMD and EV71 cases within Shawo revealed that (1) most of the cases are 3–6 years old and (2) majority of these EV71 cases attended the larger Shawo Village Daycare Center/Kindergarten, which is one of the only two daycare centers in Shawo Township. Together, these results suggest that EV71 may have possibly been transmitted through the daycare center/kindergarten in Shawo, leading to enterovirus-associated HFMD cases.

### Sequence and phylogenetic analysis of EV71 strains in Shawo

**Figure 5:**
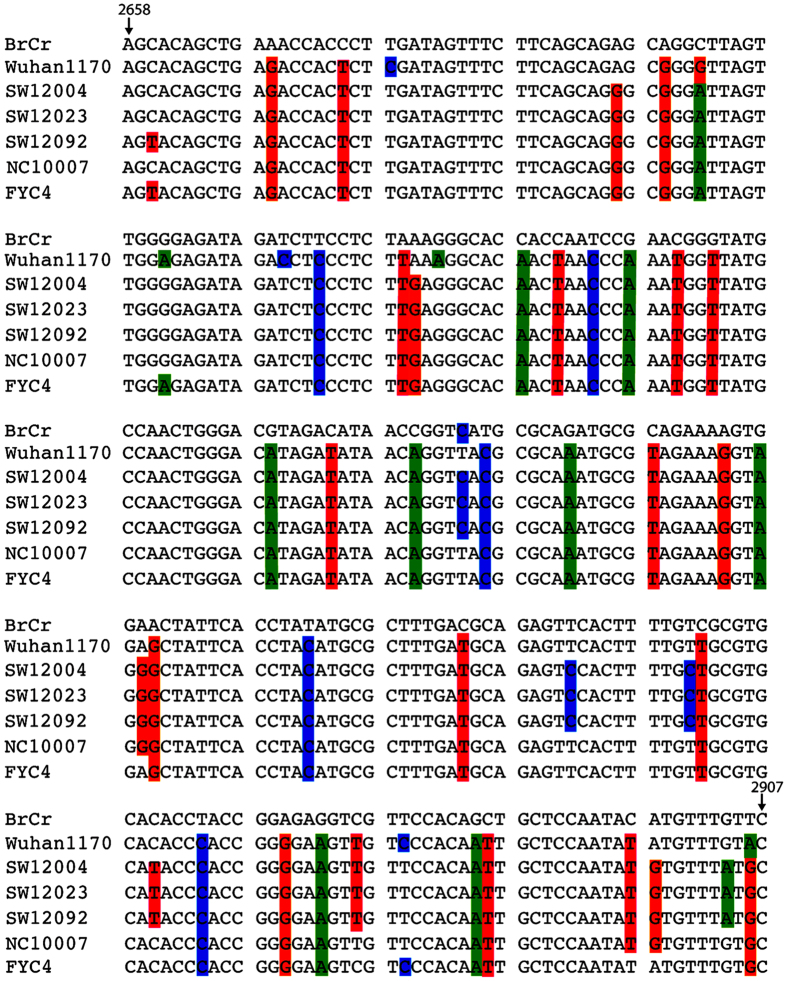
Sequence alignment of a part of the VP1 genes among EV71 strains isolated in Shawo (SW12004, SW12023, and SW12092) and the reference viruses. The reference viruses include BrCr (a genotype A strain from US in 1968), FYC4 (a genotype C4 strain from Fuyang, Anhui of China in 2008), Wuhan1170 (a genotype C4 strain isolated from the nearby city of Wuhan in the Hubei Province in 2011), and NC10007 (a genotype C4 strain isolated from the city of Nanchang in the distant Jiangxi Province in 2010). The nucleotides that are not identical among the virus strains are highlighted. Numbering starts at the 5′ terminus of the EV71 genome.

**Figure 6:**
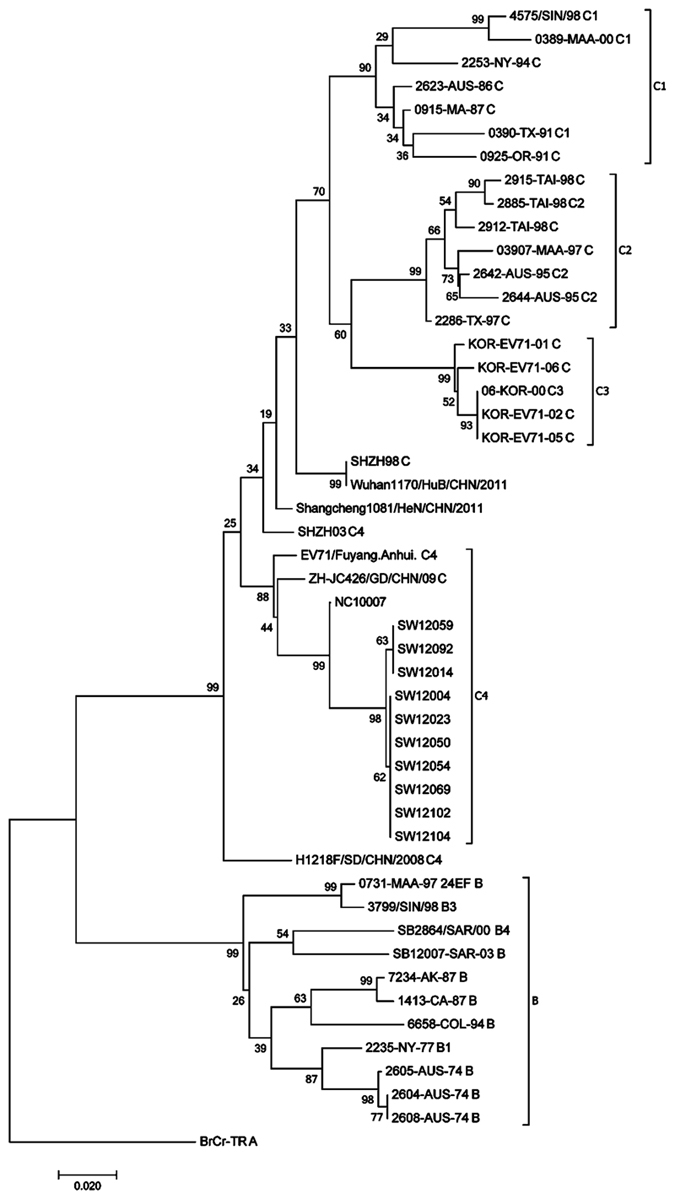
Phylogenetic analysis of the sequences of EV71 isolates from Shawo and the reference viruses. We carried out sequence comparison of different strains using alignment of the sequences of a part of the VP1 region, following the procedures described in the Methods^29^,^30^.

Viruses were isolated from ten EV71-positive samples, which represent different geographic regions of Shawo and patients from different age groups. The partial VP1 genes of EV71 were amplified by PCR, and sequenced. Figure 5 shows the Clustal W-assisted alignment^32^ of the nucleotide sequence of the partial VP1 genes of three EV71 samples from Shawo (i.e. SW12004, SW12023, and SW12092). We also included reference strains BrCr (a genotype A strain from US in 1968) and FYC4 (EV71/Fuyang.Anhui, a genotype C4 strain from Fuyang, Anhui of China in 2008). In addition, we included strain Wuhan1170 (Wuhan1170/HuB/CHN/2011, a genotype C4 strain isolated from the nearby city of Wuhan in the Hubei Province in 2011) and NC10007 (a genotype C4 strain isolated from the city of Nanchang in the distant Jiangxi Province in 2010). A phylogenetic tree was generated with the sequences of these ten isolates and reference EV71 strains from Genbank using MEGA software^33^ (Fig. 6). The analysis also included the genetic distance in the phylogenetic tree (Fig. 6). Phylogenetic analyses showed that the Shawo EV71 isolates are clustered into the same cluster of subgenotype C4 commonly found in mainland China during EV71 outbreaks in 2008^31^,^34^,^35^. Surprisingly, Shawo EV71 isolates appeared to be phylogenetically closer to the EV71 found in the distant city of Nanchang (Jiangxi Province) than to those in the nearby city of Wuhan (Hubei Province) (Fig. 6). By analyzing VP1 partial sequences, our results suggested that the Shawo isolates shared about 99.75% sequence similarity in their VP1 sequences. Furthermore, our results suggested that the Shawo isolated shared about 98%, 91%, 97%, and 83% sequence similarity with the Nanchang strain (NC10007), Wuhan strain (Wuhan1170/HuB/CHN/2011), Fuyang FYC4 strain (EV71/Fuyang.Anhui), and the genotype A strain (BrCr), respectively. These results suggest that Shawo EV71 isolates belong to the subgenotype C4 which may have originated from Nanchang and the neighboring Anhui province (city of Fuyang), the center of EV71 epidemic in 2008.

## Discussion

Our studies describe an outbreak of HFMD and epidemiological analysis in Shawo Township, a rural township in Hubei Province of China between September 1 and October 28, 2012. Our results indicate that 90% of reported HFMD cases were children aged below 9 years. In particular, the 3–6 year old age group contained the highest number of HFMD cases. Of 66 enterovirus-positive samples, 48 were EV71- positive, while 16 were CVA16- positive. This is the first study so far to investigate the epidemiology of HFMD and EV71 infection during outbreaks in Shawo Township of Hubei Province in 2012.

It is possible that our epidemiological analysis may not truly represent the distribution of HFMD and enterovirus infections as the qRT-PCR assays may have false positive or negative results. To address these issues, we designed the study and interpreted the results carefully. First, all clinical data was obtained in the Shawo People’s Clinic, which is the only regional clinic in the entire Shawo Township. As such, most patients suspected of HFMD were sent to the clinic. Second, all patients were given a thorough medical exam and clinical samples were collected. Third, for each qRT-PCR assay, three sets of primers were used to detect enteroviruses, EV71 and CVA16, and each assay was performed and repeated three times. Finally, virus samples were cultured from some of the samples positive for EV71 and their sequences were determined and analyzed. Thus, we believe that our results may accurately represent the distribution of HFMD and enteroviruses in Shawo in 2012.

To study the molecular characterization of the EV71 strains circulating in Shawo, we isolated viruses from ten EV71-positive samples and carried out sequencing analysis. A phylogenetic tree was generated with the sequences of these ten isolates and reference EV71 strains from Genbank and the analysis also included the genetic distance in the phylogenetic tree (Fig. 6). Although it is currently not known about the nature of the EV71 strains circulating in Shawo, recent studies have reported the characterization of EV71 strains in the adjacent region in the city of Wuhan of Hubei Province as well as strains circulating in neighboring provinces including Anhui and Jiangxi^29^,^30^,^35^. These results have shown that EV71 strains circulating in these areas belong to subgroup C4, with distinct nucleotide sequences with each unique geographical region.

Sequence analyses suggest that the isolated EV71 viruses may belong to the subgroup C4 and appeared to be evolutionally close to EV71 strains circulating in the distant Nanchang area (Jiangxi Province) but not to those strains in the nearby Wuhan area (Hubei province)(Fig. 6). Indeed, our analyses of the VP1 partial sequences suggested that the Shawo isolates shared about 99.75% sequence similarity in their VP1 sequences. Furthermore, our results suggested that the Shawo isolated shared about 98%, 91%, 97%, and 83% sequence similarity with the Nanchang strain (NC10007), Wuhan strain (Wuhan1170/HuB/CHN/2011), Fuyang FYC4 strain (EV71/Fuyang, Anhui), and the genotype A strain (BrCr), respectively. It is conceivable that these outbreak-causing strains may not represent native strains and might have been recently introduced to Shawo, possibly by tourists or the returning of the migrant workers to Shawo from Jiangxi Province. We note that we cannot completely rule out the possibility that these strains represent native circulating strains in Shawo because no information is currently available about the nature of EV71 strains circulating in Shawo. Further studies of the HFMD cases and EV71 strains in Shawo and the other regions of China will help reveal the distribution and epidemiology of EV71 infection and HFMD.

Enteroviruses may transmit among individuals via intra-familial and intra-community routes including attendance in schools^36^,^37^. Previous studies indicated that attending childcare or kindergarten significantly increased the seropositive rates of anti-EV71 antibodies^1^,^2^,^3^. It has also been reported that epidemics of EV71 spread from schools to the community at large^36^,^37^. In our studies, the peak of HFMD cases and EV71 infection occurs within children of 3–6 years old. These results are consistent with the previous observations that attending childcare or kindergarten represents a major route for the transmission of EV71 and the spread of HFMD^36^,^37^. Most children aged 3–6 years old attend daycare and kindergarten in China. Indeed, more than 80% of the children from 3–6 years old in our studies attended Shawo Daycare Center/Kindergarten, one of the only two kindergarten/daycare centers in the entire district. These results imply that the daycare center may play an important role in the transmission of enteroviruses within the community, although we cannot rule out other possible unidentified means of transmission among these affected children.

In China, most of the children younger than 1 year old stay at home. The children of this age group were cared for by their family members in our study based on our interviews and site visits. The few HFMD cases and EV71 infection within this age group suggest that intra-familial transmission may not represent a major route responsible for the HFMD and EV71 outbreaks in Shawo as studied in this report. The few cases of HFMD and EV71 infection among children of more than 7 years old may be explained by the notion that stronger immune responses associated with this group were due to better developed immune systems and/or pre-existing immunity as a results of prior exposure to EV71 infection^4^,^5^.

To prevent potential EV71 spread in schools, the Chinese government has recommended hand-washing, quarantine, and some other precautions as part of Public Health policy. The Shawo Kindergarten/Daycare Center has implemented these similar measures, including requesting students to wash hand frequently and asking sick children not to come to school and to stay at home. Our results highlight the potential risk of attending kindergarten/daycare center and suggest that complete implementation and strict compliance of universal precaution measures are crucial to prevent HFMD outbreak and EV71 infection.

Our preliminary studies focused on isolated EV71 virus strains, and additional studies may be carried out to analyze CVA16 virus isolated from our HFMD samples and characterize these viruses in order to study the correlation between EV71/CVA16 infections and HFMD-associated diseases. We note that all of our reported HFMD cases are benign, without any severe or fatal cases. One possible explanation is the difference in the level of virulence of enteroviruses circulating in Shawo and other regions. We note that the sequences of the Shawo EV71 strains isolated in this study were phylogenetically more close to those isolated in the distant Nanchang region (Jiangxi Province) than those isolated in the neighboring Wuhan region (Hubei Province) with distinct variations in nucleotide sequences (Figs 5 and 6)^29^,^30^,^35^. In our study, more female patients were infected with EV71 than male patients in Shawo (Fig. 4). In contrast, more male patients were infected with EV71 than female patients in Wuhan and Nanchang^29^,^30^. Furthermore, the majority of the EV71-infected patients were 3–6 years old in Shawo (Fig. 3) while the majority of the EV71-infected individuals were 1–3 years old in Nanchang^29^,^30^. These results suggest that infections by the EV71 strains circulating in Shawo, which contained distinct sequence variations, might exhibit some clinical characteristics different from those by the EV71 strains circulating in Wuhan and Nanchang^29^,^30^,^35^. Additional studies will be performed to investigate the relationships between EV71 sequence mutations and their clinical outcomes. These studies will improve our understanding of EV71 infections and HFMD outbreaks.

## Methods

### Ethics Statement

All research involving human participants was approved by the Institutional Review Board of the College of Life Sciences at Wuhan University (Wuhan, China) and that of the College of Life Sciences at Jinan University in accordance with the guidelines for the protection of human subjects. Written informed consent was obtained from each participant.

### Clinical information and specimen collection

All reported HFMD cases in Shawo were recorded by medical practitioners of the People’s Clinic of Shawo Township, following the procedures described previously^29^,^30^. Clinical surveys were collected from the clinic and used in this research project for an epidemiologic study. Throat swabs and fecal samples were also collected from each case when applicable by medical practitioners of the Shawo People’s Clinic. These samples were used in the laboratory for viral isolation and diagnosis.

We made site visits to the Shawo People’s Clinic, daycare centers, and different villages within Shawo Township. We examined the playgrounds, classrooms, and sleeping areas in the schools. We interviewed teachers regarding children’s activities during school, the school’s prevention methods towards HFMD, and information known about the cases that occurred there. In addition, we also visited the homes of some of the children and met with their parents, and interviewed physicians at the Shawo People’s Clinic.

### Viral RNA extraction from specimens and quantitative RT-PCR analysis

Viral RNA was extracted from the clinical samples using a Roche High Pure Viral RNA Extraction Kit (Roche, Germany), according to the manufacturer’s experimental protocols^29^,^30^. Reverse transcription was carried out in the presence of M-MLV Reverse Transcriptase (Promega, USA), following the previously described procedures^29^,^30^. We carried out the qRT-PCR analysis to detect the universal EV (EVU) sequence, and the EV71 and CVA16 sequences, following the procedures described previously^29^,^30^. Reactions were prepared in a 25-μL volume by using a One Step PrimeScript RT-PCR kit (BioPerfctus Technologies, Inc., Shanghai, China) and performed in a Light Cycler 480 II (Roche, Germany). All the reactions were performed in triplex.

### Sequencing and phylogenetic analyses

The sequences corresponding to the VP1 regions of EV71 viruses were amplified from the cultured virus samples using PCR, cloned, and sequenced, following the procedures described previously^29^,^30^. The GenBank accession numbers of a part of the VP1 sequences of the ten EV71 isolates reported in this study are KX276156-KX276165. Nucleotide BLASTn analysis (http://www.ncbi.nlm.nih.gov/BLAST) was used to identify related reference viruses, and the reference sequences were obtained from GenBank. We carried out pair-wise sequence alignments with the Megalign program (DNASTAR) and reference EV71 sequences from GenBank, using Clustal W program implemented with MEGA 4.0^32,33^. Phylogenetic analyses of the aligned sequences for a part of the VP1 gene were performed by the neighbor-joining method with 1000 bootstraps using MEGA 4.0, following the previously described procedures^29^,^30^.

### Data analysis

Information contained in the clinical surveys collected from the Shawo People’s Clinic was stored and filled into new surveys using the data analysis program *Epi Info* (http://wwwn.cdc.gov/epiinfo/). We carried out data analysis by calculating the frequencies of the different variables within the surveys, following the procedures described previously^29^,^30^. For certain variables some cases were omitted due to missing information included on the corresponding surveys.

## Additional Information

**How to cite this article**: Liu, M. Y. *et al*. Characterization of enterovirus 71 infection and associated outbreak of Hand, Foot, and Mouth Disease in Shawo of China in 2012. *Sci. Rep.* **6**, 38451; doi: 10.1038/srep38451 (2016).

**Publisher's note:** Springer Nature remains neutral with regard to jurisdictional claims in published maps and institutional affiliations.

## Supplementary Material

Supplementary Information

## Figures and Tables

**Figure 1:**
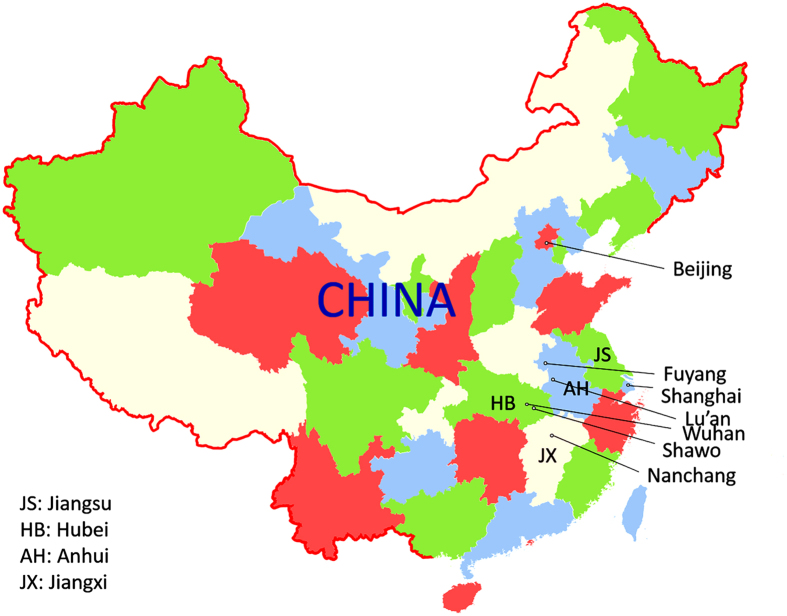
Geographic location of Shawo Township in China. The map is drawn with Microsoft PowerPoint 2013 Software and further modified and converted to TIF format in Adobe Photoshop CS5.

**Table 1:** Characteristics of different aged groups of HFMD cases in Shawo.

Age	Under 1	1–2	2–3	3–4	4–5	5–6	6–7	7–8	8–9	over 9
Case (No. and %)	0(0.0)	5(4.8)	14(13.3)	20(19.0)	19(18.1)	17(16.2)	11(10.5)	6(5.7)	8(7.6)	5(4.8)
EV71 (No. and %)	0(0.0)	1(20.0)	3(21.4)	12(60.0)	13(68.4)	11(64.7)	4(36.4)	2(33.3)	2(25.0)	0(0.0)
CVA16 (No. and %)	0(0.0)	1(20.0)	3(21.4)	2(10.0)	2(10.5)	3(17.6)	2(18.2)	1(16.7)	1(12.5)	1(10.0)
Both EV71 and CVA16 (No. and %)	0(0.0)	0(0.0)	1(7.1)	2(10.0)	2(10.5)	3(17.6)	0(0.0)	0(0.0)	0(0.0)	0(0.0)
Negative for EV71 or CVA16 (No. and %)	0(0.0)	3(60.0)	9(64.3)	8(40.0)	6(31.6)	6(35.3)	5(45.5)	3(50.0)	5(62.5)	4(80.0)
Fever (No. and %)	0(0.0)	3(60.0)	11(78.6)	16(80.0)	14(73.7)	15(88.2)	7(63.6)	5(83.3)	6(75.0)	2(40.0)
Cough(No. and %)	0(0.0)	0(0.0)	2(14.3)	7(35.0)	5(26.3)	9(52.9)	3(27.3)	3(50.0)	6(75.0)	3(60.0)
Catarrh symptoms(No. and %)	0(0.0)	4(80.0)	9(64.3)	12(60.0)	10(52.6)	10(58.8)	8(72.7)	1(16.7)	2(25.0)	2(40.0)
Oral lesion (No. and %)	0(0.0)	3(60.0)	8(57.1)	9(45.0)	9(47.4)	10(58.8)	4(36.4)	0(0.0)	1(12.5)	2(40.0)
Vesicles on hand and foot (No. and %)	0(0.0)	1(20.0)	6(42.9)	14(70.0)	10(52.6)	8(47.1)	8(72.7)	6(100.0)	7(87.5)	4(80.0)

AH: Anhui Province, HB: Hubei Province, JS: Jiangsu Province, JX: Jiangxi Province.

**Table 2:** Distribution of HFMD cases and the incidences of viral infections in different villages of Shawo.

Village	HFMD cases	EVU	EV71	CVA16	EV71/CVA16
Baotuan	5	3	2	1	0
Caopi	4	3	2	1	1
Huangshan	3	3	3	0	0
Huqiao	4	0	0	0	0
Jiajiang	4	2	2	0	0
Pailou	8	5	2	0	0
Shawo	52	30	24	9	4
Xinwan	15	13	8	1	1
Yingshan	2	1	1	0	0
Yuba	1	1	0	1	0
Zhaozhai	2	2	2	1	1
Zouma	5	3	2	2	1
