# Supplementary material for: Characterization of enterovirus 71 infection and associated outbreak of Hand, Foot, and Mouth Disease in Shawo of China in 2012
Source: Sci Rep. 2016 Dec 12;6:38451. doi: 10.1038/srep38451 (PMC5150535; doi:10.1038/srep38451)
Supplement: Supplementary Information [file srep38451-s1.pdf]

Characterization of enterovirus 71 infection and associated outbreak of Hand, Foot, and Mouth Disease in Shawo of China in 2012

Michelle Y. Liu[1,2,3#], Jin Liu[2#], Weijian Lai[1,4,5], Jun Luo[2], Yingle Liu[2], Gia-Phong Vu[3], Zhu Yang[4,5], Phong Trang[3], Hongjian Li[1]*, Jianguo Wu[2]*

[1]College of Life Sciences, Jinan University, Guangzhou, Guangdong 510632, China

[2]State Key Laboratory of Virology, College of Life Sciences, Wuhan University, Wuhan, Hubei 430072, China

[3]School of Public Health, University of California, Berkeley, CA 94720

[4]Taizhou Institute of Virology, Taizhou, Jiangsu 225300, China

[5]Jiangsu Affynigen Biotechnologies, Inc., Taizhou, Jiangsu 225300, China

[#]These authors contribute equally to this study.

*Correspondence should be addressed to:

Dr. Jianguo Wu

State Key Laboratory of Virology, College of Life Sciences, Wuhan University, Wuhan 430072, P.R. China, Tel: +86-27-68754001, E-mail: jwu@whu.edu.cn

Dr. Hongjian Li

College of Life Sciences, Jinan University, Guangzhou, Guangdong 510632, China. Tel: +86-20-85220501, Email: tlihj@jnu.edu.cn

SUPPLEMENTAL INFORMATION

Table S1. Quantitative RT-PCR detection of the EV-A71, CV-A16, and human enterovirus universal (EVU) sequences in the HFMD samples.

Number	EV-A71	CV-A16	EVU
SW12001	——[a]	——	——
SW12002	——	——	——
SW12003	——	——	——
SW12004	positive	——	positive
SW12005	positive	positive	positive
SW12006	positive	positive	positive
SW12007	positive	positive	positive
SW12008	——	——	——
SW12009	——	——	——
SW12010	——	——	——
SW12011	——	——	——
SW12012	——	positive	positive
SW12013	positive	positive	positive
SW12014	positive	——	positive
SW12015	positive	——	positive
SW12016	——	positive	positive
SW12017	positive	positive	positive
SW12018	——	——	——
SW12019	——	——	——
SW12020	positive	——	positive
SW12021	——	——	——
SW12022	——	positive	positive
SW12023	positive	——	positive
SW12024	——	——	——
SW12025	——	——	——
SW12026	——	——	——
SW12027	——	——	positive
SW12028	positive	——	positive
SW12029	positive	——	positive
SW12030	——	positive	positive
SW12031	positive	——	positive
SW12032	positive	——	positive
SW12033	——	——	——

SW12034	——	——	——
SW12035	——	——	——
SW12036	——	——	——
SW12037	——	——	——
SW12038	——	positive	positive
SW12039	——	positive	positive
SW12040	——	——	positive
SW12041	——	——	——
SW12042	——	——	——
SW12043	——	——	——
SW12044	——	——	——
SW12045	positive	——	positive
SW12046	——	——	——
SW12047	positive	——	positive
SW12048	positive	——	positive
SW12049	positive	——	positive
SW12050	positive	——	positive
SW12051	——	——	——
SW12052	positive	——	positive
SW12053	positive	——	positive
SW12054	positive	——	positive
SW12055	——	——	positive
SW12056	——	positive	positive
SW12057	——	——	positive
SW12058	positive	——	positive
SW12059	positive	——	positive
SW12060	——	positive	positive
SW12061	——	——	——
SW12062	positive	——	positive
SW12063	positive	——	positive
SW12064	——	——	——
SW12065	positive	——	positive
SW12066	——	——	positive
SW12067	positive	——	positive
SW12068	——	——	positive
SW12069	positive	——	positive
SW12070	——	——	——
SW12071	positive	——	positive
SW12072	——	——	positive
SW12073	positive	positive	positive
SW12074	positive	——	positive
SW12075	——	——	positive
SW12076	positive	——	positive

SW12077	positive	——	positive
SW12078	——	——	——
SW12079	positive	——	positive
SW12080	——	——	positive
SW12081	——	——	positive
SW12082	positive	——	positive
SW12083	positive	——	positive
SW12084	——	——	——
SW12085	positive	positive	positive
SW12086	——	——	——
SW12087	positive	——	positive
SW12088	positive	——	positive
SW12089	——	——	——
SW12090	——	——	——
SW12091	positive	positive	positive
SW12092	positive	——	positive
SW12093	——	——	——
SW12094	positive	——	positive
SW12095	positive	——	positive
SW12096	——	——	——
SW12097	——	——	——
SW12098	——	——	——
SW12099	——	——	——
SW12100	——	——	——
SW12101	positive	——	positive
SW12102	positive	——	positive
SW12103	positive	——	positive
SW12104	positive	——	positive
SW12105	——	——	——

[a] Negative;
